# Hydrological modeling of geophysical parameters of arboviral and protozoan disease vectors in Internally Displaced People camps in Gulu, Uganda

**DOI:** 10.1186/1476-072X-7-11

**Published:** 2008-03-14

**Authors:** Benjamin G Jacob, Ephantus J Muturi, Erick X Caamano, James T Gunter, Enoch Mpanga, Robert Ayine, Joseph Okelloonen, Jack Pen-Mogi Nyeko, Josephat I Shililu, John I Githure, James L Regens, Robert J Novak, Ibulaimu Kakoma

**Affiliations:** 1 Department of Medicine, William C. Gorgas Center for Geographic Medicine, Birmingham, AL, 35294, USA; 2 Center for Biosecurity Research University of Oklahoma Health Sciences Center 755 Research Parkway, Suite 520 Oklahoma City, OK, 73104, USA; 3 Human Health Division, International Centre of Insect Physiology and Ecology (ICIPE), Nairobi, Kenya; 4 Gulu University, Gulu, Uganda; 5 Mekerere Medical School, Box 7072, Kampala, Uganda; 6 University of Illinois, College of Veterinary Medicine, 2001 S Lincoln, Urbana, IL, 61801, USA

## Abstract

**Background:**

The aim of this study was to determine if remotely sensed data and Digital Elevation Model (DEM) can test relationships between *Culex quinquefasciatus *and *Anopheles gambiae *s.l. larval habitats and environmental parameters within Internally Displaced People (IDP) campgrounds in Gulu, Uganda. A total of 65 georeferenced aquatic habitats in various IDP camps were studied to compare the larval abundance of *Cx. quinquefasciatus *and *An. gambiae *s.l. The aquatic habitat dataset were overlaid onto Land Use Land Cover (LULC) maps retrieved from Landsat imagery with 150 m × 150 m grid cells stratified by levels of drainage. The LULC change was estimated over a period of 14 years. Poisson regression analyses and Moran's *I *statistics were used to model relationships between larval abundance and environmental predictors. Individual larval habitat data were further evaluated in terms of their covariations with spatial autocorrelation by regressing them on candidate spatial filter eigenvectors. Multispectral QuickBird imagery classification and DEM-based GIS methods were generated to evaluate stream flow direction and accumulation for identification of immature *Cx. quinquefasciatus *and *An. gambiae *s.l. and abundance.

**Results:**

The main LULC change in urban Gulu IDP camps was non-urban to urban, which included about 71.5 % of the land cover. The regression models indicate that counts of *An. gambiae *s.l. larvae were associated with shade while *Cx. quinquefasciatus *were associated with floating vegetation. Moran's *I *and the General G statistics for mosquito density by species and instars, identified significant clusters of high densities of *Anopheles*; larvae, however, *Culex *are not consistently clustered. A stepwise negative binomial regression decomposed the immature *An. gambiae *s.l. data into empirical orthogonal bases. The data suggest the presence of roughly 11% to 28 % redundant information in the larval count samples. The DEM suggest a positive correlation for *Culex *(0.24) while for *Anopheles *there was a negative correlation (-0.23) for a local model distance to stream.

**Conclusion:**

These data demonstrate that optical remote sensing; geostatistics and DEMs can be used to identify parameters associated with *Culex *and *Anopheles *aquatic habitats.

## Background

Flood and swamp water mosquito abundance can be predicted in real time using high resolution data through application of a dynamic hydrological model [1]. These models account for topographic variability and their control over soil moisture heterogeneity and runoff within a shed. Soil moisture levels can be associated with local mosquito biting rates on humans and entomologic inoculation rates (EIR) [2]. The probability distribution of the soil moisture deficit, i.e., statistics of topography, is generated from digital elevation model (DEM) data by using a multidirectional flow routing algorithm, which is tied to an adaptive error correction (pit infill) scheme needed for low-relief areas such as coastal plains [3]. DEM can yield several catchment hydrological variables including percent surface saturation, and total surface runoff for identification of spatial distribution of potential mosquito aquatic habitats within a catchment [4].

Monitoring water table depth (WTD) permits detection of mosquito breeding pools at very fine, sub-meter pixel, spatial scales. For example, a modeled local WTD for a given pixel of -0.2 m does not imply that the pixel is dry, merely that water may accumulate at this location. Given the variability in surface elevation and WTD, a percentage of the water table can be expected to protrude through the soil level. The shallower the local WTD for a given pixel, the greater the percentage of that pixel area can be estimated to be wet at the surface [5]. Thus, a pixel with a mean WTD, of -0.4 m can be expected to have more surface pooling than a pixel with a WTD of -1.4 m. DEM's have shown that substantial soil moisture heterogeneity exists at most scales within a catchment [6]. This fractal geometry permits such extrapolation of the pixel-to-pixel variability of local WTDs to a smaller, subpixel scale. Hoof prints, ditches, tire tracks, and natural relief can all account for the heterogeneity of elevation at the pixel level [1]. Therefore the use of local WTD allows a statistical estimation of such potentially saturated portions of the surface exploited by floodwater mosquitoes.

In this paper, we examine the distribution of mosquitoes across various IDP's camps in Gulu, Uganda through the use of high spatial resolution satellite sensor imagery and georeferenced field sampled mosquito data. The research objectives were to : a) compute Land Use Land Cover (LULC) indices; b) determine non-spatially dependent and spatially dependent ecological covariates affecting larval abundance; c) spatially filter all residuals, and, c) construct a Digital Elevation Model (DEM) that researchers can use to identify potential aquatic habitats of *Cx quinquefasciatus *and *An. gambiae *s.l.

## Methods

### Study Site

Gulu district is located in northern Uganda. The district is made up of 19 sub-counties and 4 divisions. There are 120 parishes in the rural sub-counties and 16 wards in the divisions and a total of 406 villages. Collectively these IDP camps are located between longitude 30°–32° east; latitude 02°–04° degrees north. The IDP camps are bordered by Sudan on the north, Pader District on the east, Kitgum District on the northeast and Arua District on the west. The other district borders include Adjumani on the northwest, Masindi on the south and Apac on the southeast and Nebbi on the southwest. Gulu is 332 km from Kampala. The mean annual rainfall is 1,500 mm with the monthly average rainfall varying between 1.40 mm in January and 230 mm in August. Normally the wet season extends from April to October with the highest peaks in May, August and October, while the dry season begins in November and extends to March. Survival rates per gonadtrophic cycle for *An. gambiae *s.l. averaged 0.31 during the short rains, 0.49 during the dry season and 0.78 during the long rains with low vectoral capacities due to low survival rates and a high degree of zoophily [7]. The average maximum temperature is 50°C and the minimum is 18°C. The relative humidity is high during the wet season and low in the dry season. Vegetation within the IDP camps is classified by Lang lands (1974) as of intermediate savannah grassland. The vegetation type is characterized by open canopy of trees 10 m to 12 m high and underlying grasses of 80 cm high. The soil of Gulu IDP camps consists of ferruginous sandy with a high percentage of sandy soils and, therefore, susceptible to erosion. Due to the sandy nature, the soil has low water retention capacity and high rate of water infiltration. Flooding, soil amendment and crop phenology affect the population dynamics of *Anopheles *and *Culex *species [8].

**Figure 1:**
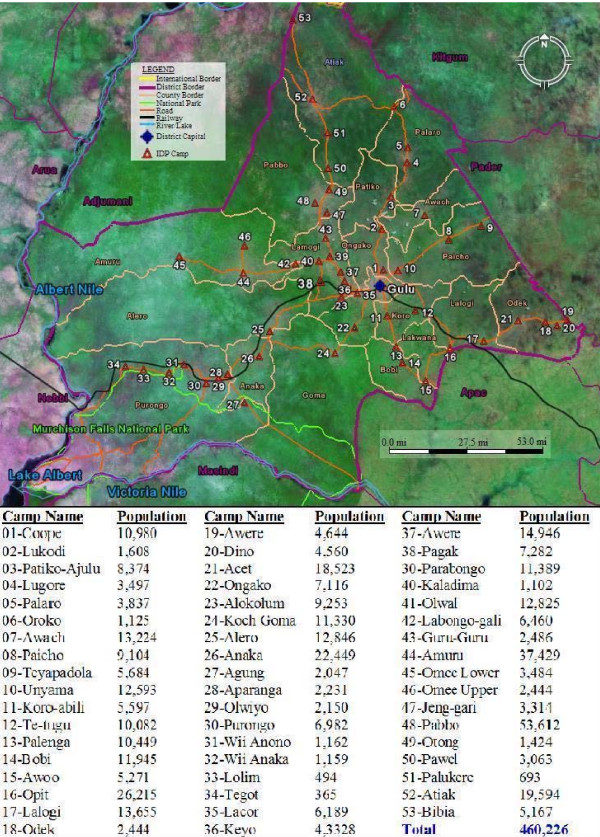
**Base map of Internally Displaced Peoples (IDP) camps in Gulu study area**. This a map of IDP camps sites and different land cover types. Also included are the population statistics for each IDP camp.

Gulu is located in a zone protracted civil conflict region. Consequently of the total population of 462,581, approximately 92% (416,322) have settled temporarily in the IDP camps, where relative security can be provided. Therefore, overcrowding is common the IDP camp. IDP camp communities rely heavily on the surrounding forest and vegetation cover for fuel wood, and on subsistence faming to supplement humanitarian assistance. The houses are the grass thatched houses. The utilities include makeshift toilets. Water pumps are the only source for clean drinking water in the camps. Multiple artificial water storage containers are found within close proximity (e.g. bucket) to many houses. The average household size is six. The IDPS are composed of the indigenous population of Gulu District, the Acholi. Many of the IDP camps are found along the main roads, trading centers and its suburbs (Figure 1).

### Larvae Sampling

In Gulu IDP camps, 65 aquatic habitats were randomly selected and mapped using a CSI-Wireless differentially corrected global positioning systems (DGPS) Max receiver. Each aquatic habitat was sampled throughout May 2006 to June 2006. All water bodies were inspected for mosquito larvae using standard dipping techniques with a 350 ml dipper to collect the mosquito larvae and pupae [9]. *Culex *and *Anopheles *mosquito larvae were identified morphologically using the keys at the Human Health Division, International Centre of Insect Physiology and Ecology (ICIPE) in Nairobi, Kenya. Culicine and Anopheline mosquitoes were identified to specific species using the identification key constructed by Gillies and Coetzee [10].

### Larval habitat characterization

Environmental variables recorded for each aquatic habitat were water depth, pH, water surface area, distance to the nearest house, canopy coverage, surface debris coverage, algal coverage, emergent plant coverage, turbidity, habitat type, and substrate type. Distance to the nearest house was measured with a tape when it was shorter than 100 m. When the distance exceeded 100 m, it was estimated visually. The distance to the nearest house was categorized into 7 classes (e.g., 1: 0–100 m, 2: 101–200 m, and so on, and 7 for distances greater than 600 m). Canopy cover was defined as the amount of terrestrial vegetation and other objects in the habitat. Emergent plants included both aquatic and immersed terrestrial vegetation. Plant coverage of a habitat was measured in percentage of water surface covered by placing a square frame (1 m^2^) with 100 grids (10 cm^2^) above the habitat. Turbidity was measured by placing water samples in glass test tubes and holding against a white background, and classified into 4 levels: clear, low, medium, and high turbidity. The habitat types included animal footprints, pond (water area larger than 50 m^2^), stream pool, puddle (water area less than 50 m^2^), water tank, and tire track. Substrate types were classified into muddy, sandy, gravel with soil, and artificial substrate without soil (e.g., concrete or brick).

### Habitat base-mapping

Each georeferenced *An. gambiae *s.l. and *Cx. quinquefasciatus *aquatic habitat from the study site were entered into a Vector Control Management System^® ^(VCMS) (Clarke Mosquito Control Products, Inc. 159 N. Garden Avenue. Roselle, IL 60172) database. VCMS supported the mobile field data acquisition in each village through a Microsoft PocketPC™. All two-way, remote synchronizing of data, geocoding, and spatial display were processed using the embedded GIS Interface Kit™ that was built using Earth Systems Research Institute (ESRI, Redlands, CA, USA) MapObjects™ 2 technology. The VCMS database plotted and updated the DGPS ground coordinates of larval habitats and supported exporting data in a GIS shape file format.

### Remote Sensing Data

Visible and near-infrared (NIR) information ranging from 0.45–0.90 μm from the Landsat 7 ETM+ image obtained July 28, 2005 and Thematic Mapper™ (TM) from July 15,1991 were used to create a multitemporal LULC change dataset in Erdas *Imagine *V8.7^® ^(Atlanta, GA, USA). The TM image of the study site data consisted of four spectral bands with a spatial resolution of 30 m for bands 1–4. The Universal Transverse Mercator (UTM) Zone 36S datum and WGS-84 projection was used for all spatial datasets.

### Satellite data classification and land cover analysis

The satellite data were classified using the Iterative Self-Organizing Data Analysis Technique (ISODATA) unsupervised routine in ERDAS *Imagine *V9.1^® ^(Atlanta, GA, USA). This approach to classification has been used widely in the identification of land covers and mosquito habitats associated with intermediate hosts and disease vectors [11-15]. The image was classified into 6 categories residential, industrial, crops, forest, water bodies, and grass. These categories were aggregated according to the following categorization.

1) Urban: Urban classes encompassed the following categories: man-made physical infrastructures. This land cover class included residential, industrial, transportation, and communications/utilities. This class also included the livestock buildings and barns for cows, pigs, and chickens.

2) Non-urban: Vegetated (mainly natural vegetation classes), woodland (trees > 5 m tall; 25%–75% canopy). This land cover class included mixed woodland, grass lands and old field with mixed scattered trees.

3) Water: This class included permanent bodies of water such as lakes, streams and essentially any open water area or area covered by water the majority of the time. This class also included areas with hydrophilic vegetation, wetlands with a high water table and areas interspersed with channels or pools of open water.

### Stratifying an urban grid

New maps for this study site were generated from QuickBird data using the DGPS ground coordinates in ArcInfo 9.1^® ^(ESRI, 2005a, CA, USA). The QuickBird image bands were acquired May, 2006. QuickBird multispectral products provide four discrete non-overlapping spectral bands covering a range from 0.45 to 0.72 μm with an 11-bit collected information depth [16]. A 150 m × 150 m orthogonal grid cell was overlaid on a QuickBird image of the study site with the purpose of sampling and providing a comparison technique for LULC and mosquito aquatic habitats.

The study area was stratified based on the level of drainage present within each grid cell. A grid cell was classified as well-drained if functional (e.g. clear of debris or vegetation at the time of observation) engineered drainage systems were present and no standing water was visible, or if the grid cell was located on a slope and no standing water was visible. A grid cell was classified as poorly drained if it was located in a depression or valley and had either no drainage systems, or the drainage systems were blocked with debris or vegetation.

A unique identifier was assigned to each grid cell. The level of house spacing, road types and networks, community water sources, and access to utilities within grid cells also were noted. Information contained in the census of the study site and District Development Reports, as well as, environmental descriptions from previous field and topographical maps were used to assist with the stratification process.

To determine the number of samples appropriate for collection in the IDP camps villages, we evaluated *Anopheles *and Culex larval count data from multiple villages in Kenya. We expected the larval count in the IDP camp mosquito aquatic habitats to follow a Poisson distribution, as was the case in the Kenyan villages [17-19]. Therefore, we used the mean count and standard deviations on the log number of larval counts collected in the Kenyan villages to determine sample size requirements. We applied a sampling intensity formula for determining the number of samples to collect when randomly sampling from an infinite population n = (ts/E)^^2 ^where t = t value (t = 2), s = the standard deviation of ln count values observed in Kenyan villages (s = 0.889) and E is desired half-width of the confidence interval around the mean expressed in same units as standard deviation (E = ln(1.25) [20]. Applying this formula and assuming larval production is similar for mosquito habitats in Kenya and Uganda, we determined 65 samples were required. We overlaid vector image of the sampling scheme (grid cell) with the LULC raster image to identify areas of interest within each polygon (grid cell) of the sampling scheme. All potential aquatic larval habitat sites were identified, and data relative to species composition and abundance, predators, water quality and environmental parameters were collected longitudinally.

### Data Analysis Strategy

Field data parameters were analyzed using SAS 9.1.3 ^® ^(SAS inc. Carey, NC, USA). The differences in larval counts among habitat types and LULC changes were compared by an ANOVA test. Where significant differences were observed, the means were separated by Tukey's HSD test. LULC was examined for each sample unit at the study site to determine the amount of the land cover change between 1991 and 2005. A chi-square test was performed to assess the relationship between LULC change and strata. Poisson regression analyses were used to determine the relationship between *An. gambiae *s.l. and *Cx. quinquefasciatus *larval counts and the measured habitat characteristics. Larval data were log-transformed before analyses to normalize the distribution and minimize standard error. All the covariates were tested for multicollinearity using partial F test in SAS and no problematic correlations were found.

### Spatial analyses

We used spatial methods to identify the trend in the ecological dataset. Moran Coefficients (MC), a product moment correlation coefficient type of spatial autocorrelation index provides a technique for indexing spatial autocorrelation. Subsequently, we detrended the data using spatial autocorrelation analysis. The most straightforward hypothesis with which to test statistical significance of the MC assumes spatial autocorrelation is zero. The MC may be tested using analytical expectations and variances based largely on the neighborhood structure assumed in a spatial weighting matrix [21]. The general formula for computing Moran's *I *is [22]:

$$I=\frac{N\sum_{i=1}^{N}\sum_{j=1}^{N}w_{ij}z_{i}z_{j}}{\sum_{i=1}^{N}\sum_{j=1}^{N}w_{ij}\sum_{i=1}^{N}z_{i}^{2}}$$

Where

n = number of values to be taken into account

y_i/j _= value at location i and j

*w*_*ij *_is the weight at distance *d*, that is, *w*_*ij *_= 1 if point *j *is within distance class *d *from point *i*, else *w*_*ij *_= 0;

*z*'s are deviations (i.e., *z*_*i *_= *y*_*i*_*-y*_*mean *_for variable *y*),

*W *is the sum of all the weights. The summation is done for all *i *not equal to *j*.

The MC was generated on the residual of the detrended data in ArcGIS. The MC and high/low clustering was measured using Getis-Ord General G preformed from the functions provided in Arc Toolbox. The spatial autocorrelation analyses measured feature similarity based on sample locations, species and larval aquatic habitat density values simultaneously in the study site. The tool evaluated whether the pattern expressed was clustered, dispersed, or random. An MC value near +1.0 indicated clustering of either high or low-density mosquito larvae measures while an index value near -1.0 indicated dispersed measures of mosquito density. A Z-score was calculated for assessing whether the observed clustering or dispersion was statistically significant or not.

### Spatial filtering analyses

From results of the MC and Getis spatial test for clustering it was indicated that there was spatial autocorrelation in the anopheline larval data. We filtered the spatial components of the *An. gambiae *s.l. larval data. SAS PROC GENMOD was also used to build a Poisson model with a non-constant, gamma-distributed mean (i.e., negative binomial model). Spatial filtering seeks to transform a variable containing spatial dependence into one free of spatial dependence by partitioning the original georeferenced attribute variable into two synthetic variates: a spatial filter variate capturing latent spatial dependency that otherwise would remain in the response residuals, and a nonspatial variate that is free of spatial dependence [23]. Both positive and negative spatial autocorrelation eigenvectors were selected by a stepwise negative binomial regression procedure. Because eigenvectors are mutually orthogonal and uncorrelated in a linear model, a normal approximation stepwise regression was executed. This procedure confirmed that both positive and negative spatial autocorrelation eigenvectors were needed to describe the geographic distribution of the *An. gambiae *s.l. larval counts. This initial screening also was performed because Generalized Linear Model (GLM) estimation involves weighting schemes that corrupt, to some degree, these properties of orthogonality and un-correlation.

To expand the inferential basis with a random effect, a Generalized Linear Mixed Model (GLMM) was used to account for latent non-spatial residual correlation. The GLMM estimation was computed using SAS PROC NLMIXED. Rather than switching from a Poisson to a negative binomial probability model, the GLMM was extended to account for latent non-spatial correlation effects as well as to allow inferences to be drawn for a much wider range of geographic sampling configurations than those utilized by employing a GLMM. The GLMM included a random effect, which is specified here as a random intercept that was assumed to be normally distributed with a mean of zero, a constant variance and zero spatial autocorrelation. This varying intercept term compensated for the nonconstant mean associated with a negative binomial GLMM specification. All parameters except the intercept were treated as single-valued, while the intercept was treated as a distribution of values.

A Box-Cox type of power transformation was employed in the study site for normal approximation analysis purposes so that the frequency distributions of the *An. gambaie *s.l. larval counts better mimicked a bell-shaped curve. We used the spatial filter construction methodology transformation procedure as proposed by Griffith [23] that depends on the eigenfunctions of matrix (**I **- **11**^T^/n)**C**(**I **- **11**^T^/n) – where **I **denotes the identity matrix, **1 **is an n-by-1 vector of ones, and T denotes matrix transpose – a term appearing in the numerator of the MC spatial autocorrelation index. According to Griffith [24], the first eigenvector, **E**_1_, is the set of numerical values that has the largest Moran's *I *achievable by any set for the spatial arrangement defined by the geographic connectivity matrix **C**. The second eigenvector is the set of values that has the largest achievable MC by any set that is uncorrelated with **E**_1_. The third eigenvector is the third such set of values. And so on. This sequential construction of eigenvectors continues through **E**_n_, the set of values that has the largest negative MC achievable by any set that is uncorrelated with the preceding (n-1) eigenvectors.

To identify spatial clusters that can be uncovered with spatial filtering, Thiessen polygon surface partitionings were generated for the study site in order to construct geographic neighbor matrices, each denoted by matrix **C**, which also was used in spatial autocorrelation analysis. Entries in matrix **C **were 1 if two points share a common Thiessen polygon boundary and 0 otherwise; the diagonal was coded 0. Next, the linkage structure for each surface was edited in order to remove unlikely geographic neighbors (i.e., pairs of sample locations sharing a common Thiessen polygon boundary, but whose separation is too great) [25]. Attention was restricted here to those map patterns associated with at least a minimum level of spatial autocorrelation, which for implementation purposes was defined by |MC_j_/MC_max_| > 0.25, where MC_j _denoted the jth value and MC_max _the maximum value of MC. This threshold value allowed two candidate sets of eigenvectors to be considered, one for substantial positive and one for substantial negative spatial autocorrelation.

### Digital Elevation Model (DEM)

A DEM of the study area was downloaded from seamless United States Geological Survey (USGS, March 17^th^, 2007). The use of the DEM for determining environmental parameters for establishing a predictive model has been proven by different studies on the ecology of malaria vectors including *An. gambiae *s.l and the impact of landscape on their populations and malaria transmission [4]. The DEM was constructed based on a contour map of 1:50,000. The purpose of DEM construction was to extract topographic variables that were associated with mosquito larval habitat formation, such as elevation, flow accumulation, flow direction and stream order. Wetness index or topographic index represents land surface moisture content. It was calculated as ln(*A/TanB*) where *A *was the upslope contributing area and *TanB *was the local slope. Parameters *A *and *TanB *were derived using a multiple flow-direction algorithm.

The Stream Raster Grid was generated in ArcGIS. The advantage of using flow distance-to-stream rather than simple distance-to-stream is that flow distance takes flow direction and landscape profile into consideration. Euclidian distance-to-nearest hydrological body was calculated as the distance from a grid cell to a stream grid cell defined by a Stream Raster Grid. Flow distance-to-stream may affect availability of the aquatic habitat and is calculated as the distance from a grid cell moving downstream to a stream grid cell defined by the Stream Raster grid. The Terrain Analysis Using DEM (TauDEM) in ArcGIS was used to retrieve these parameters. A three-dimensional model of the study area was constructed based on the DEM using ArcScene extension of ArcGIS.

## Results

A total of 65 aquatic habitats belonging to five habitat types were identified in the study area and inspected for mosquito larvae. These included ditches (47.7%), canals (35.4%), seeps (9.21%), pools (6.22%) and tire tracks (1.54%). Anopheline larvae were present in 31 habitats and 16 (24.6%) of these habitats had only anophelines. Culicine larvae were found in 34 habitats and 15 (23.1%) of these habitats had only culicines. Chi square analysis indicated that coexistence anopheline and culicine larvae in the same habitats was not greater than would be expected by chance alone (χ^2 ^= 0.12, p = 0.73). The mean number of anopheline larvae collected per sample was 6.46 ± 1.44 while that of culicine was 14.6 ± 6.04 per sample. Canals were the most important habitats in anopheline productivity (ANOVA, F = 3.10, p = 0.02) whereas among culicines, there was no significant difference in larval density among habitat types (ANOVA, F = 1.58, p = 0.19). The overall larval abundance for anopheline was 6.45.

The *An. gambiae *s.l. and *Cx. quinquefasciatus *larval densities collected in diverse habitat types among the well and the poorly drained strata are represented in Table 1. Although there was no significant difference in larval densities among habitats located within the well and the poorly drained strata, 5 habitat types were identified in the poorly drained strata compared to 3 in the well drained strata. The importance of a particular habitat type varied amongst strata. (Table 1).

**Table 1:** *Anopheles gambiae *s.l. and *Culex. quinquefasciatus *larval density in diverse habitat types identified in the IDP camps study site

Drainage	Habitat type	*Anopheles spp*.	*Culex spp*.
Well drained	Canal	0.30 ± 0.30	0.80 ± 0.42
	Ditch	0.09 ± 0.09	0.18 ± 0.12
	Seep	0.50 ± 0.50	0.50 ± 0.50
	Total	0.22 ± 0.14	0.48 ± 0.20

Poorly drained	Canal	0.54 ± 0.29	1.77 ± 0.67
	Ditch	0.35 ± 0.17	1.50 ± 0.60
	Pool	0.00 ± 0.00	2.25 ± 2.25
	Seep	0.00 ± 0.00	23.5 ± 23.2
	Tire track	0.00 ± 0.00	0.00 ± 0.00
	Total	0.33 ± 0.12	3.71 ± 2.21

The total number of pixels in the LULC classification was 27.0 km^2 ^of which 15.3 km^2 ^of land cover showed change between 1991 and 2005. The percentage of overall LULC change for 14 years in the Gulu IDP camps was 56.6 %. The main land cover category in the Gulu IDP camps was non-urban to urban, which included about 71.5 % of the land cover.

Accordingly, land cover changes between 1991 and 2005 were analyzed in ArcInfo 9.1^® ^and mapped. Significantly higher number of aquatic habitats positive for *Culex *and *Anopheles *larvae were observed in LULC change sites than in non-LULC change sites (Table 2). The most frequent LULC change positive for *Culex *(F = 2.27 df = 1, 64, *P *= 0.05) and *Anopheles *(F = 2.32 df = 1, 64, *P *= 0.05) larvae was non-urban to urban.

**Table 2:** Summary of total aquatic habitats showing the proportion of site positive for *Culex quinquefasciatus *and *Anopheles gambiae *s.l. aquatic habitats in LULC change sites in the IDP camps study site

LULC change	n	*Cx. quinquefasciatus *larvae/20 dip	*An. gambiae *s.l. larvae/20 dip
Non-urban to -urban	34	6.31	2.46
Urban to non-urban	6	0.74	0.22
Urban to water	4	1.11	0.49
Water to urban	0	0.37	0.02
Non-urban to water	2	1.25	0.81
Water to non-urban	1	0.82	0.24
Non-change	18	3.97	2.22

Of the 13 ecological variables that were entered into the model, two were found to be significant predictors of larval abundance. *Cx. quinquefasciatus *larvae was negatively associated with emergent vegetation while *An. gambiae *s.l was negatively associated with shade. Turbidity was also a significant positive predictor for both species (Table 3).

**Table 3:** Poisson regression results, with levels of significance with percent of total deviation of the field and satellite parameters for the IDP camps study site for abundance and distribution *An. gambiae *s.l. and *Cx. quinquefasciatus *larval mosquitoes

Mosquito Species	Variable	Final	Beta	R^2^
*An. gambiae*	Shade	< 0.001	-1.37	16.2
	Turbidity	< 0.001	2.58	15.3
	Pseudo-R^2^			31.8
*Cx. quinquefasciatus*	Emergent vegetation	< 0.001	-1.77	13.6
	Turbidity	< 0.001	1.58	13.0
	Pseudo-R2			26.6

The measures of clustering of *An. gambaie *s.l. and *Cx. quinquefasciatus *aquatic habitats are reported in Table 4. In the analyses, significant *Z*-values had a probability being caused by random chance of less than 0.05

**Table 4:** Moran's *I *and the General G statistics with corresponding Z-values for mosquito density by species and instar, total density by species, and total density for all species.

**Larval Instar**	**Species**	**Moran's *I***	**Z**	**General G**	**Z**
1st instar	-----	0.31	4.18*	0.03	5.29*
2^nd ^instar	------	0.02	0.48	0.02	2.55*
3^rd ^instar	*An. gambiae *s.l	0.07	1.03	0.02	1.57
4^th ^instar	*An. gambiae *s.l	0.03	0.64	0.01	1.57
1^st ^instar	------	0.11	1.57	0.02	0.75
2^nd ^instar	-------	0.07	1.05	0.01	-0.63
3^rd ^instar	*Cx. quinquefasciatus*	0.07	1.03	0.13	10.9*
4^th ^instar	*Cx. quinquefasciatus*	0.00	0.13	0.01	-0.78

Significant (p < 0.05)

---- Individuals not identified to species

Estimation results from SAS PROC GENMOD for all models appear in Table 5. In each case both positive and negative spatial autocorrelation eigenvectors were selected by the stepwise negative binomial regression procedure. Positive and negative spatial autocorrelation spatial filter component pseudo-R^2 ^values are reported in Table 5.

**Table 5:** Poisson spatial filtering model results for *An gambiae *s.l. mosquito counts in the IDP camp study site

Spatial Statistics	L3	L4
SF: # of eigenvectors	5	2
SF: MC	0.671	0.092
SF pseudo-R^2^	0.192	0.128
Positive SA SF: # of eigenvectors	3	1
Positive SA SF: MC	0.586	0.943
Positive SA SF pseudo-R^2^	0.131	0.081
Negative SA SF: # of eigenvectors	1	1
Negative SA SF: MC	-0.352	-0.537
Negative SA SF pseudo-R^2^	0.048	0.067
Deviance statistic	1.171	1.145
Dispersion parameter	0.544	1.089

SF denotes spatial filter

SA denotes spatial autocorrelation

A pseudo-R^2 ^is the squared correlation between observed and GLM-predicted

GLMM estimation results from SAS PROC NLMIXED appear in Table 6. These spatial autocorrelation components suggest the presence of roughly 12% to 28% redundant information in the *An. gambiae *s.l. larval count samples.

**Table 6:** Poisson SF GLMM random effects results for *An. gambiae *s.l. larval mosquito counts in the IDP camp study site

Statistics	L3	L4
Mean	0.056	0.083
Standard deviation	0.489	0.733
MC	-0.055	0.014
Pseudo-R^2^	0.935	0.916

GLMM denotes generalized linear mixed model

MC denotes the Moran Coefficient

A DEM model was generated in ArcGIS. The range of the elevation in the DEM had a minimum value of 996 m with a maximum value of 1,132 m. The slope of the *An. gambiae *s.l. aquatic habitats was 0.171%. The slope of the *Cx. quinquefasciatus *was 0.006%. There is a significant positive correlation for *Cx quinquefsaciatus *aquatic habitat count and slope (0.24) while for *An. gambiae *aquatic habitat count and slope there was a negative correlation (-0.23) for a local model based on distance to stream.

## Discussion

In IDP camps the highest numbers of aquatic larval habitats observed were in the poorly drained strata. In earlier studies in East African urban regions many mosquito aquatic habitats were observed in the poorly drained strata, suggesting that drainage does affect habitat development at some level [26]. Canal and seeps produced the highest number of *Cx. quinquefasciatus *and *An. gambiae *s.l. habitats for all LULC change sites in which aquatic habitats were present. Ditch habitats had the least larval density, for both species. The most common locale for mosquito aquatic habitats was in LULC sites was non-urban to urban. Jacob et al. [27] reported similar findings in the study sites for *An. gambaie *s.l. aquatic habitats in the Kisumu and Malindi study sites.

The regression results indicated that counts of *An. gambiae *s.l. and *Cx. quinquefasciatus *were negatively associated with shade and emergent vegetation, respectively. In addition both species were positively associated with turbidity. Floating and emergent vegetation can obstruct mosquito oviposition and also reduce the amount of sun light reaching the aquatic habitat resulting in low water temperatures [28]. This interferes with microbial growth that forms the main diet for mosquito larvae, and increases both the larval development time and probability of contact with predators [29]. Gimnig et al. [30] found increasing *An. gambiae s.l*. larvae densities with increasing turbidity while Muturi et al. [28] found that the production of *Cx. quinquefasciatus *was favored in highly turbid water.

In the spatial analyses, the positive value for MC indicates that clustering of high and/or low larval habitat density sites for *An. gambiae *s.l. is likely and positive values for the general G statistic suggest that high larval density sites is clustered. General G analysis identifies the same areas as having significant clusters of high larval densities of *An. gambiae *s.l. larvae. Jacob et al. [31] report detecting positive spatial autocorrelation in urban *An. gambiae *s.l. mosquitoes. However, inconsistent results between MC and general G values for *Cx. quinquefasciatus *aquatic habitats measures and totals suggest larval densities are not consistently strongly clustered. Effectively controlling *Cx. quinquefasciatus *in the study site may require a more widespread approach than targeting larval "hot spots". Several key factors in arboviral transmission are known to vary across urban regions including the dominant enzootic vectors, the relationships between vector abundance and land use and differences in the composition of host communities that can, in turn, influence mosquito habitat preferences [32].

In the spatial filtering analyses, positive and negative spatial autocorrelation eigenvectors were selected using stepwise negative binomial regression. The larval *Anopheles *mosquito counts contained hidden negative spatial autocorrelation that is masked by positive spatial autocorrelation. Because the MC are asymptotically normally distributed, MC may fail to detect hidden negative spatial autocorrelation in highly heterogeneous environments though all of the visual and conventional numerical evidence suggests the presence of positive spatial autocorrelation [23].

The inclusion of a random effects term had little impact upon the resulting spatial filters. The spatial filters obtained with the Poisson spatial filtering model and GLMM analyses were almost identical for the study site. The spatial dependency in the models suggests both negative and positive components are present suggesting the presence of redundant information in the *An. gambiae *larval data. Redundant information may be attributed to the locational arrangements of sample points which may cause observations to be dependent, rather than independent, moving data analysis away from the classical statistical independence model [25].

The DEM found that *An. gambiae *s.l larval abundance was negatively associated with distance from the stream. In contrast, *Cx quinquefasciatus *larval abundance was positively associated with distance from the stream. The numerous open-sun lit pools that form at the edge or slightly upland from the stream may provide ideal larval habitats for *An. gambiae *s.l. [30]. The positive association between *Culex quinquefasciatus *and distance from the stream is biologically plausible given its preference for eutrophic aquatic habitats [33,34]. Aquatic habitats further away from the stream are likely to be rich in organic matter than those closer to the stream because they are least likely to be diluted by surface run-off from the stream.

*Cx quinquefasciatus *aquatic habitats were more prevalent in valley bottoms than on hills in the study area. In the valley bottom aquatic habitats can occur in streams and adjacent swamps. Minakawa et al. [35] found that surface runoff from uphill springs and groundwater seepage commonly form larval habitats in swamp margins at the valley bottoms.

In conclusion, 56.6% of LULC change for the urban Gulu study site in 14 years contributed to changes in abundance, and distribution of *Cx. quinquefasciatus *and *An. gambiae *s.l. aquatic habitats. There were more LULC changes than non-LULC changes in the study site. In LULC change sites, the highest percent of aquatic habitat positive for *Culex *and *Anopheles *larvae was in non-urban to urban land cover change sites. In the Poisson regression analyses,*Cx. quinquefasciatus *larvae abundance was negatively associated with emergent vegetation while *An. gambiae *s.l was negatively associated with shade. A cluster analyses revealed that high density *An. gambaie *s.l. aquatic habitats have a strong tendency to be aggregated in the study site. The spatial filter analyses described the full range of all possible mutually orthogonal map patterns present in the *An. gambiae *s.l larval data. These spatial autocorrelation components suggest the presence of roughly 12% to 28% redundant information in the *An. gambiae *s.l. larval count samples. The DEM of land surface topography and hydrological networks accounted for mosquito larval abundance for both species. The sign and magnitude of the association of modeled surface wetness and species abundance appear to be a function of mosquito species biology and overall abundance. Modelling and forecasting floodwater mosquito larval species using newer GIS software applications and fine resolution satellite data will enable local public health agencies to institute control measures before the mosquitoes emerge as adults, and their role as transmitters of disease comes into play.

## Competing interests

The author(s) declare that they have no competing interests.

## Authors' contributions

BGJ conceived the study and led the drafting of this manuscript; EJM, EXC helped analyze the non-spatial analyses; JG conducted the spatial analyses: EM and RA provided descriptive statistics of the study site. JO, PN and JS supervised the field data collection and helped analyze the data; JG, RJN and JLR provided expertise on mosquito habitats in urban environments and contributed to the interpretation of results; IK is the principal investigator of the study. All authors interpreted the results and wrote the paper.
